# Investigations on the dissolution behavior of silicon in aqueous HF-HClO_4_-mixtures†

**DOI:** 10.1039/d5ra00859j

**Published:** 2025-05-13

**Authors:** Ann-Lucia Neumann, André Stapf, Nils Schubert, Niklas Zomack, Edwin Kroke

**Affiliations:** a Technische Universität Bergakademie Freiberg, Department of Chemistry, Physics and Biosciences, Institute of Inorganic Chemistry Leipziger Str. 29 D-09599 Freiberg Germany Edwin.Kroke@chemie.tu-freiberg.de; b Center of Efficient High-Temperature Material Conversion, Technische Universität Bergakademie Freiberg Winklerstr. 5, D-09599 Freiberg Germany

## Abstract

Wet chemical etching processes are an essential part of silicon treatment in the photovoltaic and semiconductor industry. A commonly used system is HF-HNO_3_. In order to avoid NO_*x*_-formation, silicon can also be etched with HF-(HCl)-Cl_2_-mixtures. Thorough investigations into perchloric acid indicate that even Si-H terminated surfaces are inert against this very strong oxidizing agent.

## Abstract 2

Thorough investigations into perchloric acid indicate that even Si–H terminated surfaces are inert against this very strong oxidizing agent.

## Graphical abstract

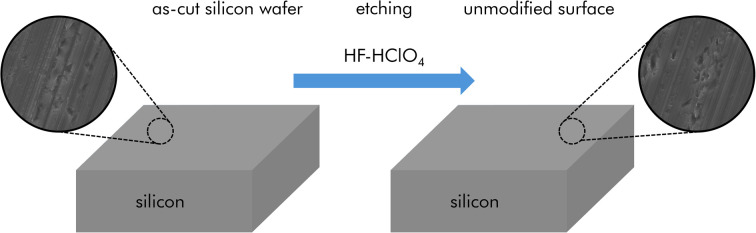

Silicon is the most important raw material in the photovoltaic and semiconductor industry.^1^ During the production of various silicon devices, silicon surfaces must be cleaned, etched and structured using wet chemical etching processes, which makes wet chemical etching an essential part of silicon processing.^2^ In general, during the chemical dissolution of silicon in aqueous solutions, the surface atoms are oxidized first and then complexed to form water-soluble species namely hexafluorosilicate ([SiF_6_]^2−^) in fluoride containing acid solutions (eqn 1.a and 1.b).^3^ This requires an oxidizing agent with a standard redox potential of at least *E*^0^ > + 0.7 V.^4^

1.a Si + (*n* − *m*) *h*^+^_VB_ → Si^*n*+^ + *m* e^−^_CB_ (*n* ≤ 4; *m* ≤ 4; *n* > *m*)

1.b Si^*n*+^ +6 HF → [SiF_6_]^2−^ + 6 H^+^ + (4 − *n*) e^−^_CB_

*h*^+^_VB_ = hole in the valence band, e^−^_CB_ = electron in the conduction band.

Silicic acid or silicate anions ([(HO)_4–*n*_SiO_*n*_]^*n*–^) with *n* = 0–4 are formed in alkaline solutions (eqn (2)).^5^

2 Si + (4 − *n*) H_2_O + *n* OH^−^ → [(HO)_4−*n*_SiO_*n*_]^*n*−^ + 2 H_2_

The treatment in alkaline solutions, *e.g.* potassium hydroxide (KOH) or sodium hydroxide (NaOH) and additives, is well known to cause anisotropically structured silicon surfaces.^5^ In contrast, isotropic removal is generally achieved by using acidic mixtures containing hydrofluoric acid (HF) and suitable oxidizing agents.^2^ The protons (in water and/or OH^−^) are the oxidizing species in alkaline solutions forming H_2_.^2,5,6^ In both cases, acidic and alkaline etching, hydrogen terminated Si surfaces are formed.^7,8^

The etching system based on hydrofluoric and nitric acid (HNO_3_) mixtures is primarily used for etching silicon in photovoltaics, due to the high etching rates (1 HF (49%): 21 HNO_3_ (70%) – 13.8 μm min^−1^)^9^ that can be observed with this system.^10,11^ Thus, cooling of the reaction mixture may be required and nitrogen oxide (NO_*x*_) containing exhaust gases as well as nitrate (NO_3_^−^) containing waste solutions are generated.^12^ Therefore, alternative oxidizing agents such as ammonium peroxodisulfate ((NH_4_)_2_S_2_O_8_), ozone (O_3_) and hydrogen peroxide (H_2_O_2_) were tested, but these solutions have a significantly lower reactivity towards silicon.^13–17^ Compared to these mixtures, bromine (Br_2_) and chlorine (Cl_2_) containing aqueous HF solutions show higher etching rates (HF-HCl-Cl_2_ – 0.63 μm min^−1^; HF-Br_2_ – 4.0 μm min^−1^).^18,19^ Chlorine was thus found as a suitable oxidizing agent for silicon, *e.g.* for saw-damage removal and spray texturing of monocrystalline silicon solar cells.^20^ In addition to chlorine, chlorine-oxygen acids could also serve as oxidizing agents, since the standard redox potential of all chlorine-oxygen acids is sufficient to oxidize the silicon surface atoms.^21^ Additionally, solutions of chlorine-oxygen acids should be easier to handle and to quantify than chlorine gas.

In general, there are four chlorine-oxygen acids: hypochlorous acid (HClO), chlorous acid (HClO_2_), chloric acid (HClO_3_) and perchloric acid (HClO_4_).^22^ Perchloric acid is the most stable chlorine oxygen acid, which is commercially available in pure form and has a standard redox potential of *E*^0^ for ClO^−^_4_/Cl^−^ = + 1.38 V at pH = 0.^22,23^ Accordingly, this acid was considered to be a suitable and promising oxidizing agent for silicon and therefore we analyzed the dissolution behavior of silicon wafers in aqueous HF-HClO_4_ solutions.

In the experiments, the same silicon material (mono-DW(100), p-doped) was used in each case and the various experiments were carried out at room temperature at a constant stirring speed. The composition of the etching mixture was varied, resulting in 42 test points of a ternary test plan (Fig. S1†). Three experiments were carried out at each test point to determine an average removal rate. The etching rates of the aqueous HF-HClO_4_ solutions were determined for a concentration of HF in the range from 0.00 mol l^−1^ to 28.92 mol l^−1^ and HClO_4_ in the range from 0.00 mol l^−1^ to 11.71 mol l^−1^, resulting in average removal rates up to 0.019 μm min^−1^ (Fig. S1†). Thus, the observed etching rates of these solutions are very low compared to the HF-HCl-Cl_2_ system (0.63 μm min^−1^).^18^

The wafer fragments were weighed before and after etching in HF-HClO_4_ solutions, so that a mass loss can be determined. Here, it is assumed that silicon is removed equally over the surface and both sides of the silicon wafer pieces. This mass loss results in a difference in thickness, which is then used to calculate the etching rate. The analytical balance has an error range of 0.0006 g for differential weighing, which means that the calculation of the etching rates with a mass difference of ≤0.0006 g is affected by errors. Since the mass differences of all wafer fragments are ≤0.0006 g, the determined removal rates of the HF-HClO_4_-mixtures are therefore within the error range. Consequently, the removal rates of the aqueous HF-HClO_4_ solutions are presumably based on measurement errors. To further verify this statement, four long-term experiments were carried out with four different compositions across the range of aforementioned concentrations and an etching period of one week. This resulted in mass differences of ≤0.0002 g, which is also within the error range of the balance. It can accordingly be assumed that no silicon has been removed from the surface.

If no silicon is removed, then no hexafluorosilicate should be present in the solution. To determine whether silicon had dissolved, the etching solutions were analyzed before and after etching using ^19^F-NMR (nuclear magnetic resonance) spectroscopy. As expected, both spectra of the analyzed HF-HClO_4_ solutions show only one signal for hydrofluoric acid at −165 ppm before and after etching, which means that the formation of hexafluorosilicate at −130 ppm was not detected (Fig. S3†).

Since there is no hexafluorosilicate in the solution, no oxidation of the silicon surface should have taken place. Therefore, treated silicon surfaces were analyzed by DRIFTS (diffuse reflectance infrared fourier transform spectroscopy) to investigate the different bonding states of the silicon surface atoms. To analyze the oxidation step of silicon in different solutions, the change in the Si-H absorption band of an H-terminated silicon surface at approximately 2100 cm^−1^ was considered (Fig. 1a)). If an H-terminated wafer is treated with HNO_3_ solution (c(HNO_3_) = 15.44 mol l^−1^), oxygen is incorporated into the Si-Si backbonds, which leads to a shift of the Si–H bands to higher wavenumbers (Fig. 1 c)).^13^ This oxygen insertion is generally only possible with oxidizing agents that contain oxygen, like H_2_O_2_,^24^ ozone,^25^ or HNO_3_.^2^ In contrast, if an H-terminated silicon wafer is treated with chlorinated water (c(Cl_2_-water) = 0.10 mol l^−1^), the Si–H bonds are cleaved and the Si–H absorption band disappears (Fig. 1d)).^17^ Similar results were recently obtained with bromine containing aqueous solutions.^19^ When using a perchloric acid solution (c(HClO_4_) = 10.54 mol l^−1^), both reaction paths are conceivable because of the presence of both chlorine and oxygen in the oxidizing agent. Since only the unchanged absorption band of the H-terminated silicon surface can be observed in the DRIFT spectrum, neither oxygen is incorporated into the rearward Si-Si bonds nor does cleavage of the Si–H bond occur (Fig. 1b)).

**Fig. 1:**
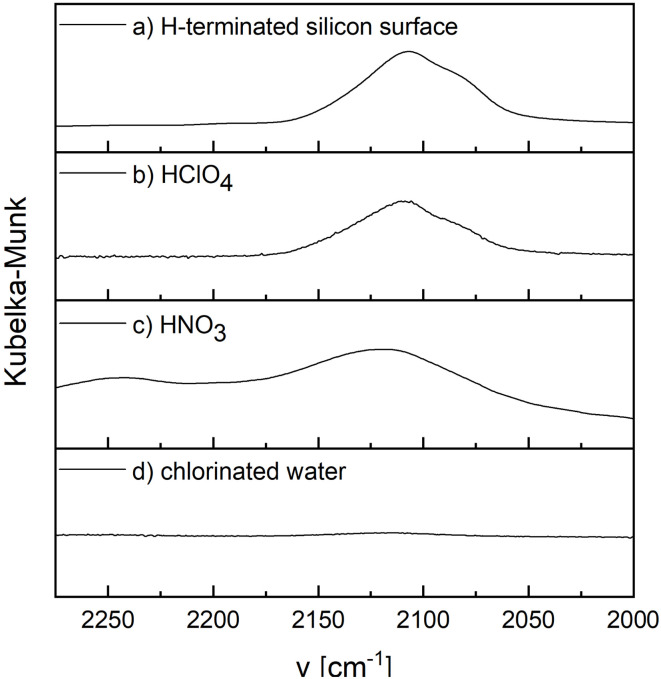
DRIFT spectra of a (a) H-terminated silicon surface treated with (b) 10.54 mol l^−1^ HClO_4_ for 15 min, (c) 15.44 mol l^−1^ HNO_3_ for 1 min and (d) 0.10 mol l^−1^ chlorinated water for 1 min. The spectra were measured at a temperature of 20 °C.

There are two different possibilities for the attack and oxidation of Si–H-terminated silicon surfaces.^2,26^ One is based on reactions of the Si–H surface moieties as observed *e.g.* in chlorine or bromine containing solutions (Fig. 1d and 2).^17–19^ This results in replacement of the hydrogen atoms, while the Si–Si-back bonds remain intact. An alternative and more frequently observed reaction behavior is the formation of Si–O–Si units in the subsurface region without initial removal of the Si–H groups, which is typical for the standard etching mixtures containing nitric acid, ozone or hydrogen peroxide.^2,24,25^ The oxidizing species in these solutions seem to be able to insert holes into the valence band of surface silicon atoms followed by insertion of oxygen atoms into the Si-Si back bonds (Fig. 1c and 2). Similar oxidation mechanisms were discussed in case of oxidation of Si–H terminated surfaces in air and native oxide formation in other oxygen containing environments.^27^ The Si–H surface groups are still present in these cases, as indicated by DRIFT spectra showing Si–H vibrations and signals which are shifted to higher wave numbers above 2150 cm^−1^. This has been discussed in terms of the bond dissociation energies of Si–Si (226 kJ mol^−1^ for crystalline silicon) and Si–H bonds (293–376 kJ mol^−1^),^28^ although kinetic aspects are much more important than thermodynamic aspects such as bond strengths.

**Fig. 2:**
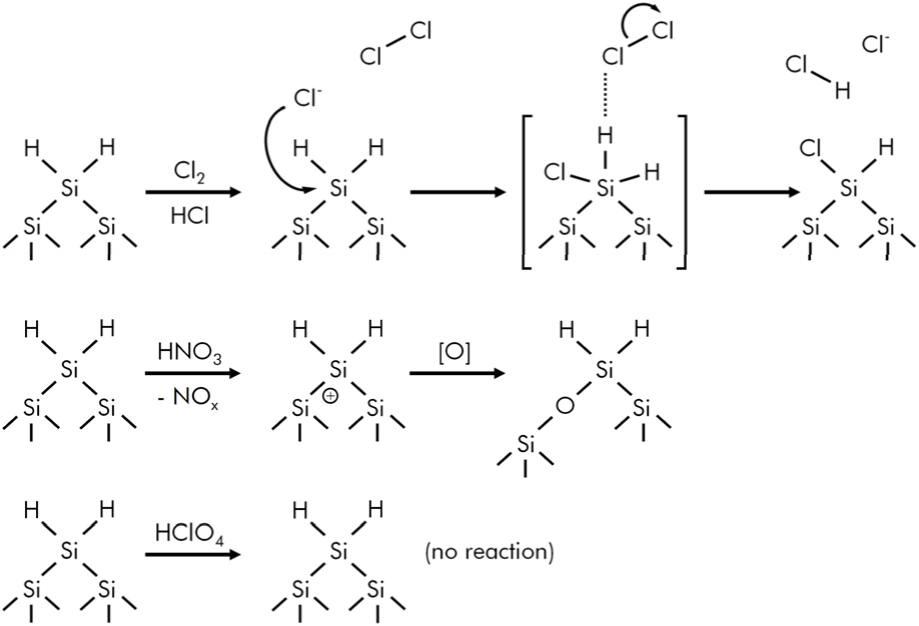
Top: suggested reaction behavior of Si–H terminated silicon surfaces in chlorine containing solutions, which explains the experimental results shown in Fig. 1d; middle: typical reaction behavior of Si–H surfaces in solutions containing oxidation agents such as nitric acid (*i.e.* NO_3_^−^ and derived species such as NO_2_^−^, NO^+^, NO_*x*_*etc.*) or other oxygen based oxidation agents (Fig. 1c); bottom: perchloric acid neither reacts with the Si–H surface nor does it induce a hole injection and/or oxygen insertion into the Si–Si-back bonds due to its chemical inertness.

As mentioned above, it is known that using an oxidizer with a high redox potential is not a sufficient condition for a high etching rate. Bond dissociation energies and redox potentials are thermodynamic data, but the etching rate is largely governed by kinetics. For the oxidation of silicon, according to ref. 4, reagents with redox potentials above +0.7 V are required. However, the kinetic barriers to either attack the Si-H bonds or to oxidize the Si–Si back bonds are too high for ClO_4_^−^.

The perchlorate ion is well known to be kinetically stable in most cases. One of the referees pointed out, that in organic electrochemistry, *e.g.* voltammetric studies in organic solvents, lithium perchlorate or tetraalkylammonium perchlorate salts are routinely used as supporting electrolytes.^29^ Despite of its oxidizing power it is reducible at cathodes only in the presence of appropriate catalysts (*e.g.*, Pt, Fe).^30^ Perchlorates are also used for electropolishing, electromachining and as electrolytes in lithium and magnesium batteries, for example.^30^

However, “the perchlorate ion is a treacherous ally”.^31^ Frost diagrams indicate that the ClO_4_^−^ is clearly the strongest oxidizing agent in acidic as well as in basic solutions, when compared to other ClO_*x*_ and ClO_*x*_^−^ species. This is also reflected in solid compounds with perchlorate anions and oxidizable counter ions such as metals with oxidizable ligands or ammonium ions. Although initial reaction rates are usually slow, fast and explosive decompositions have been reported upon mechanical action, heat, or static electricity causing very disastrous accidents. An example is the explosion a rocket fuel plant in 1988 in Nevada, where ammonium perchlorate detonated with the intensity of an earthquake,^32^ and another example is the explosion of a perchlorate salt of a lanthanide complex.^33^

Thus, while the oxidation potentials in acidic and basic solutions increase in the order Cl_2_ < ClO^−^ < ClO_2_^−^ < ClO_3_^−^ < ClO_4_^−^, the reaction rates observed in dilute solution are often in the opposite order, *i.e.* ClO_4_^−^ < ClO_3_^−^ < ClO_2_^−^ ∼ ClO^−^ ∼ Cl_2_.^31^ Peroxoanions of the heavier halogens, however, tend to react very fast and in the order ClO_4_^−^ < BrO_4_^−^ < IO_4_^−^.

In summary, the studies on the dissolution behavior of silicon in aqueous HF-HClO_4_ solutions show that there is no silicon removal, as the silicon surface atoms are not oxidized and no hexafluorosilicate is formed. Despite the strong oxidizing potential of perchloric acid, the reaction with silicon – even if it is Si–H-terminated – is kinetically completely inhibited, thus no reaction with the surface can be detected. This underlines the well known inertness of the highly symmetric and very weakly nucleophilic behavior of ClO_4_^−^ ions in many other instances, *e.g.* in electrochemistry and electrolytes containing it.

## Experimental section

Caution! Etching experiments with hydrofluoric acid must be performed in an HF-approved fume hood with HF-resistant laboratory equipment. Due to its physicochemical properties, hydrofluoric acid poses a significant health hazard.^34,35^ Hydrogen fluoride is only slightly dissociated in water, allowing HF molecules to penetrate the lipid layers of the skin and reach deep tissue. There, it disrupts calcium metabolism, causing extremely painful and poorly healing wounds. Calcium gluconate is administered as an antidote to prevent severe tissue damage and systemic toxicity. Perchloric acid also poses significant risks if handled incorrectly.^36^ Diluted, cold solutions with concentrations of up to 73% are not very dangerous, but very acidic. The risk increases with rising temperature: Above room temperature, continuous heat input can cause water to evaporate, increasing the acid concentration and significantly enhancing the oxidizing power of the perchlorate ion. Handling perchloric acid in the presence of organic compounds or in organic solvents is particularly critical, as it carries a high risk of explosion.^31–33^ Additionally, perchlorate ions can inhibit the uptake of iodide by the thyroid gland, which may lead to health issues upon repeated exposure. HF-HClO_4_ mixtures combine the hazards of both substances and require extreme caution. The addition of water to concentrated perchloric acid releases heat, which can lead to the release of hydrofluoric acid.

To prepare the etching solutions, different amounts of hydrofluoric acid (50%, Honeywell), perchloric acid (70%, Merck) and deionized water (DI) were added to 50 ml PFA beakers using a graduated pipette. The volume of each etching solution was 40 ml. The etching experiments were carried out at room temperature and the solutions were constantly mixed using a magnetic stirrer (250 rpm). The silicon wafer fragments (mono-DW(100), Woongjin, p-doped) were dipped into the solutions using PTFE tweezers. Three silicon wafer fragments per solution were etched in succession for 20 min each. After etching, the fragments were removed from the solution and rinsed with deionized water.

Determination of etching rates: the equations and a schematic drawing are given in the ESI†.

NMR spectroscopy: NMR spectra were acquired with a Bruker Avance III 500 MHz spectrometer. The measurements were carried out at a temperature of 20 °C. A sample of the etching solutions was taken before and after etching (*t* = 1557 min) of a wafer fragment.

DRIFT: a Thermo Fisher Scientific Nicolet iS50 FT-IR spec-trometer with Smart Collector Avatar accessory was used to investigate the silicon surfaces. Each silicon wafer fragment was etched for 240 s in a HF-HNO_3_ solution (c(HF) = 10.7 mol l^−1^, c(HNO_3_) = 2.8 mol l^−1^) to generate an H-terminated surface. The fragments were then rinsed with deionized water and dried with pressurized air. The wafer fragments were then dipped in an oxidation solution for 15 min (c(HClO_4_) = 10.54 mol l^−1^) or 1 min (c(HNO_3_) = 15.44 mol l^−1^) or 1 min (c(Cl_2_-water) = 0.10 mol l^−1^). After dipping in the respective oxidation solution, rinsing with deionized water and drying with pressurized air, DRIFT spectra of the wafer fragments were measured. The measurements were performed at a temperature of 20 °C. The spectra were obtained with a scan number of 128 and a data interval of 0.964 cm^−1^. Kubelka–Munk was used as the format.

## Data availability

Further details and all primary data obtained during the experiments and analysis methods are given in the experimental section and the ESI.†

## Author contributions

A.-L. N.: investigation, data curation, formal analysis, methodology, validation, visualization, writing – original draft; A. S.: conceptualization, funding acquisition, methodology, project administration, supervision, writing – review & editing; N. S.: investigation, methodology, validation, supervision, writing – review & editing; N. Z.: investigation, methodology, validation, supervision, writing – review & editing; E. K.: conceptualization, funding acquisition, project administration, resources, supervision, writing – review & editing.

## Conflicts of interest

There are no conflicts to declare.

## Supplementary Material

RA-015-D5RA00859J-s001
