# Supplementary material for: Investigations on the dissolution behavior of silicon in aqueous HF-HClO4-mixtures
Source: RSC Adv. 2025 May 13;15(20):15796–800. doi: 10.1039/d5ra00859j (PMC12070797; doi:10.1039/d5ra00859j)
Supplement: RA-015-D5RA00859J-s001 [file RA-015-D5RA00859J-s001.pdf]

Supplementary Information (SI) for RSC Advances.
This journal is © The Royal Society of Chemistry 2025

Supplementary Information

Investigations on the dissolution behavior of silicon in aqueous HF-$HClO_4$-mixtures

Ann-Lucia Neumann [a], André Stapf [a], Nils Schubert [a], Niklas Zomack [a], Edwin Kroke[*a,b]

Ternary test plan and etching rates of the aqueous HF-$HClO_4$ solutions 2

Determination of etching rates 2

^{19}F-NMR spectra 3

Data of the etching experiments 4

[a] Technische Universität Bergakademie Freiberg; Department of Chemistry, Physics and Biosciences; Institute of Inorganic Chemistry; Leipziger Str. 29, D-09599 Freiberg, Germany. E-Mail: Edwin.Kroke@chemie.tu-freiberg.de

[b] Center for Efficient High-Temperature Material Conversion, Technische Universität Bergakademie Freiberg, Winklerstr. 5, D-09599, Freiberg, Germany

Ternary test plan and etching rates of the aqueous HF-$HClO_4$ solutions

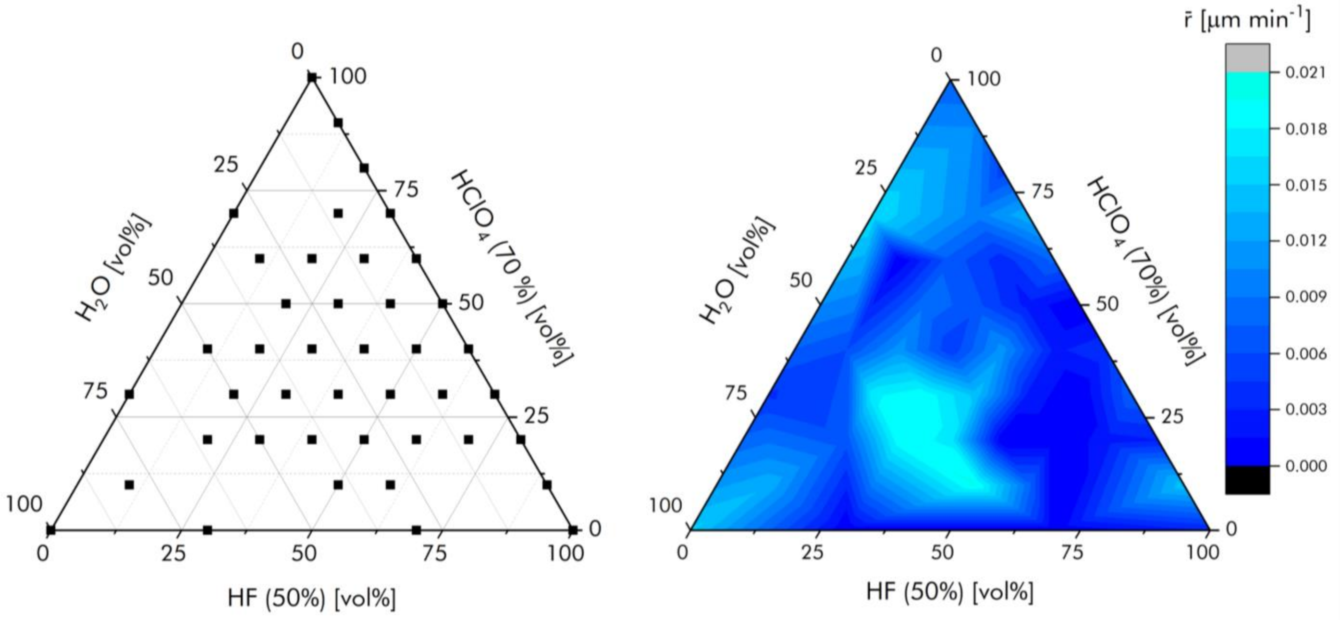

Fig. S1: Ternary test plan of the test points carried out (black squares) of the HF-$HClO_4$-H_2O system (left). A total of 42 different etching solutions were used for the etching experiments. Contour diagram with average etching rates (in µm min^{-1}) for the HF-$HClO_4$-H_2O system (right). The experiments were performed at a temperature of 20 °C and a stirring speed of 250 rpm. The etching time was 20 min.

Determination of etching rates

The Precisa XB 120 A analytical balance is used to determine the masses of the wafers. To calculate the thickness of the whole wafer, its mass, density and surface area are required (Eq. S1). The surface area of the wafer fragment results from the mass before etching, the density and the thickness of the whole wafer (Eq. S2). The differential weighing of the wafer fragments before and after etching results in a mass loss, which results in a difference in thickness (Eq. S3). The etching rate per side can be determined in µm min^{-1} using the difference in thickness and the etching time (Eq. S4).

(1) $$d_0 = \frac{m_{wafer} \cdot 10^4}{\rho_{Si} \cdot A_{wafer}}$$

(2) $$A = \frac{m_0}{\rho_{Si} \cdot d_0 \cdot 10^{-4}}$$

(3) $$\Delta d = d_0 - d_1 = d_0 - \frac{m_1 \cdot 10^4}{\rho_{Si} \cdot A} = d_0 - \frac{m_1 \cdot 10^4}{\rho_{Si} \cdot \left(\frac{m_0}{\rho_{Si} \cdot d_0 \cdot 10^{-4}}\right)} = d_0 \cdot \left(1 - \frac{m_1}{m_0}\right)$$

(4) $$r = \frac{\Delta d}{2 \cdot t}$$

d_0 = thickness of the whole wafer [μm], m_{wafer} = mass of the whole wafer [g], ρ_{Si} = density of silicon [g/cm^3], A_{wafer} = surface area of the whole wafer [cm^2], A = surface area of the wafer fragment [cm^2], m_0 = mass of the wafer fragment before etching [g], Δd = thickness difference [μm], d_1 = thickness of the wafer fragment after etching [μm], m_1 = mass of the wafer fragment after etching [g], r = etching rate per side [μm min^{-1}], t = etching time [min]

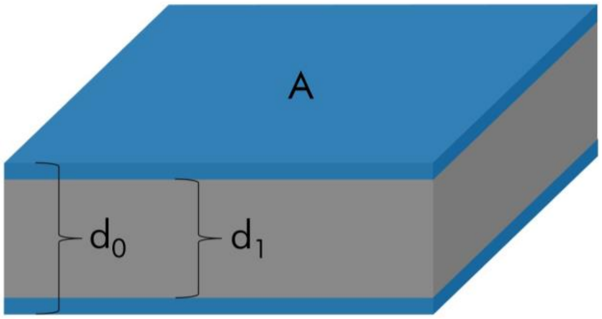

Fig. S2: Schematic drawing of a wafer fragment to visualize the difference in thickness for calculating the etching rate.

^{19}F-NMR spectra

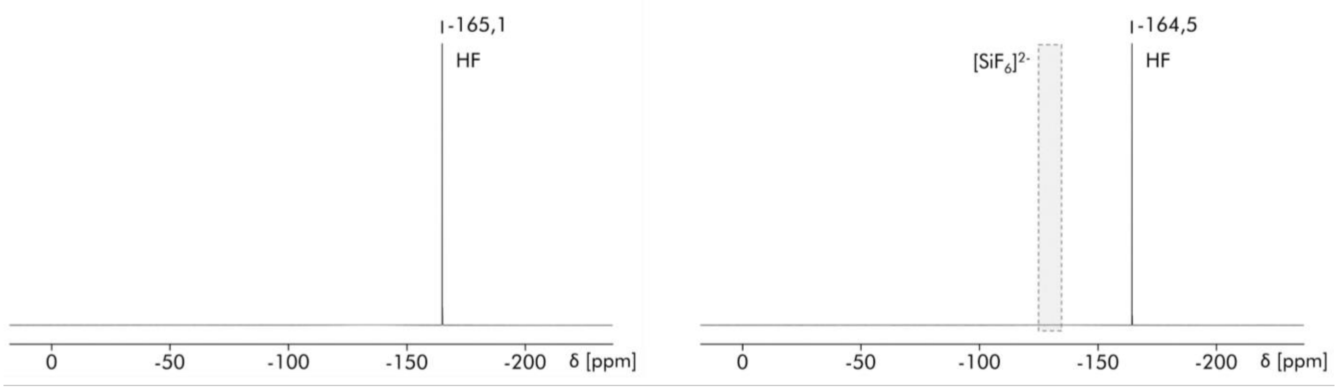

Fig. S3: ^{19}F-NMR spectra of a HF-$HClO_4$ solution (c(HF) = 11.6 mol/l, c($HClO_4$) = 2.3 mol/l) before (left) and after etching (right) of a silicon wafer fragment. The spectra were measured at a temperature of 20 °C and the etching time was 1557 min.

Data of the etching experiments

number	c(HF) [mol/l]	c($HClO_3$) [mol/l]	m_0 [g]	d_0 [µm]	m_1 [g]	Δm [g]	Δd [µm]	t [min]	r [µm min^{-1}]	$\bar{r}$ [µm min^{-1}]
1-1	5.78	2.34	0.0696	180.382	0.0695	0.0001	0.259	20	0.006	0.006
2-1	20.24	2.34	0.0752	178.397	0.0753	-0.0001	-0.237	20	-0.006	0.002
2-2			0.0673	178.397	0.0672	0.0001	0.265	20	0.007	
2-3			0.0543	178.397	0.0549	-0.0006	-1.971	20	-0.049	
3-1	5.78	8.19	0.0883	180.382	0.0882	0.0001	0.204	20	0.005	0.009
3-2			0.0800	180.382	0.0797	0.0003	0.676	20	0.017	
3-3			0.0712	180.382	0.0711	0.0001	0.253	20	0.006	
4-1	14.46	5.85	0.0979	180.382	0.0982	-0.0003	-0.553	20	-0.014	0.000
4-2			0.0638	180.382	0.0640	-0.0002	-0.565	20	-0.014	
4-3			0.0625	180.382	0.0631	-0.0006	-1.732	20	-0.043	
5-1	11.57	4.68	0.0848	180.382	0.0849	-0.0001	-0.213	20	-0.005	0.011
5-2			0.0823	180.382	0.0817	0.0006	1.315	20	0.033	
5-3			0.0724	180.382	0.0726	-0.0002	-0.498	20	-0.012	
6-1	8.67	7.02	0.0810	180.382	0.0809	0.0001	0.223	20	0.006	0.003
6-2			0.0922	180.382	0.0921	0.0001	0.196	20	0.005	
6-3			0.0659	180.382	0.0664	-0.0005	-1.369	20	-0.034	
7-1	17.35	3.51	0.0792	180.382	0.0794	-0.0002	-0.456	20	-0.011	0.000
7-2			0.0881	180.382	0.0888	-0.0007	-1.433	20	-0.036	
7-3			0.0795	180.382	0.0797	-0.0002	-0.454	20	-0.011	
8-1	8.67	8.19	0.0710	180.382	0.0711	-0.0001	-0.254	20	-0.006	0.013
8-2			0.0553	180.382	0.0550	0.0003	0.979	20	0.024	
8-3			0.0623	180.382	0.0621	0.0002	0.579	20	0.014	

number	c(HF) [mol/l]	c($HClO_3$) [mol/l]	m_0 [g]	d_0 [µm]	m_1 [g]	Δm [g]	Δd [µm]	t [min]	r [µm min^{-1}]	$\bar{r}$ [µm min^{-1}]
9-1	20.24	3.51	0.0707	180.382	0.0706	0.0001	0.255	20	0.006	0.007
9-2			0.0584	180.382	0.0582	0.0002	0.618	20	0.015	
9-3			0.0716	180.382	0.0716	0.0000	0.000	20	0.000	
10-1	11.57	7.02	0.0701	180.382	0.0700	0.0001	0.257	20	0.006	0.008
10-2			0.0497	180.382	0.0498	-0.0001	-0.363	20	-0.009	
10-3			0.0807	180.382	0.0804	0.0003	0.671	20	0.017	
11-1	17.35	4.68	0.0794	180.382	0.0795	-0.0001	-0.227	20	-0.006	0.004
11-2			0.0734	180.382	0.0732	0.0002	0.492	20	0.012	
11-3			0.0541	180.382	0.0541	0.0000	0.000	20	0.000	
12-1	5.78	9.36	0.0797	180.382	0.0799	-0.0002	-0.453	20	-0.011	0.005
12-2			0.0573	180.382	0.0572	0.0001	0.315	20	0.008	
12-3			0.0652	180.382	0.0651	0.0001	0.277	20	0.007	
13-1	23.13	2.34	0.0660	180.382	0.0663	-0.0003	-0.820	20	-0.020	0.003
13-2			0.0526	180.382	0.0525	0.0001	0.343	20	0.009	
13-3			0.0800	180.382	0.0800	0.0000	0.000	20	0.000	
14-1	11.57	5.85	0.0695	180.382	0.0697	-0.0002	-0.519	20	-0.013	0.002
14-2			0.0960	180.382	0.0961	-0.0001	-0.188	20	-0.005	
14-3			0.0635	180.382	0.0634	0.0001	0.284	20	0.007	
15-1	14.46	4.68	0.0547	180.382	0.0548	-0.0001	-0.330	20	-0.008	0.003
15-2			0.0587	180.382	0.0586	0.0001	0.307	20	0.008	
15-3			0.0780	180.382	0.0780	0.0000	0.000	20	0.000	
16-1	8.67	5.85	0.0718	180.382	0.0715	0.0003	0.754	20	0.019	0.006
16-2			0.0565	180.382	0.0567	-0.0002	-0.639	20	-0.016	
16-3			0.0551	180.382	0.0553	-0.0002	-0.655	20	-0.016	

number	c(HF) [mol/l]	$c(HClO_3)$ [mol/l]	m_0 [g]	d_0 [µm]	m_1 [g]	Δm [g]	Δd [µm]	t [min]	r [µm min^{-1}]	$\bar{r}$ [µm min^{-1}]
17-1	14.46	3.51	0.0490	180.382	0.0492	-0.0002	-0.736	20	-0.018	0.002
17-2			0.0820	180.382	0.0820	0.0000	0.000	20	0.000	
17-3			0.0604	180.382	0.0603	0.0001	0.299	20	0.007	
18-1	5.78	7.02	0.0582	180.382	0.0583	-0.0001	-0.310	20	-0.008	0.007
18-2			0.0773	180.382	0.0771	0.0002	0.467	20	0.012	
18-3			0.0511	180.382	0.0510	0.0001	0.353	20	0.009	
19-1	17.35	2.34	0.0640	180.382	0.0643	-0.0003	-0.846	20	-0.021	0.000
19-2			0.0824	180.382	0.0824	0.0000	0.000	20	0.000	
19-3			0.0572	180.382	0.0573	-0.0001	-0.315	20	-0.008	
20-1	5.78	5.85	0.0696	180.382	0.0698	-0.0002	-0.518	20	-0.013	0.009
20-2			0.0739	180.382	0.0739	0.0000	0.000	20	0.000	
20-3			0.0530	180.382	0.0527	0.0003	1.021	20	0.026	
21-1	14.46	2.34	0.0548	180.382	0.0549	-0.0001	-0.329	20	-0.008	0.000
21-2			0.0501	180.382	0.0501	0.0000	0.000	20	0.000	
21-3			0.0713	180.382	0.0713	0.0000	0.000	20	0.000	
22-1	8.67	4.68	0.0436	180.382	0.0437	-0.0001	-0.414	20	-0.010	0.005
22-2			0.0642	180.382	0.0640	0.0002	0.562	20	0.014	
22-3			0.0821	180.382	0.0823	-0.0002	-0.439	20	-0.011	
23-1	11.57	3.51	0.0696	180.382	0.0697	-0.0001	-0.259	20	-0.006	0.014
23-2			0.0729	180.382	0.0730	-0.0001	-0.247	20	-0.006	
23-3			0.0533	180.382	0.0528	0.0005	1.692	20	0.042	
24-1	5.78	3.51	0.0752	180.382	0.0752	0.0000	0.000	20	0.000	0.017
24-2			0.0575	180.382	0.0574	0.0001	0.314	20	0.008	
24-3			0.0515	180.382	0.0510	0.0005	1.751	20	0.044	

number	c(HF) [mol/l]	c($HClO_3$) [mol/l]	m_0 [g]	d_0 [µm]	m_1 [g]	Δm [g]	Δd [µm]	t [min]	r [µm min^{-1}]	$\bar{r}$ [µm min^{-1}]
25-1	8.67	2.34	0.0590	180.382	0.0591	-0.0001	-0.306	20	-0.008	0.019
25-2			0.0642	180.382	0.0638	0.0004	1.124	20	0.028	
25-3			0.0657	180.382	0.0653	0.0004	1.098	20	0.027	
26-1	2.89	10.54	0.0417	178.397	0.0415	0.0002	0.856	20	0.021	0.010
26-2			0.0506	178.397	0.0505	0.0001	0.353	20	0.009	
26-3			0.0500	178.397	0.0500	0.0000	0.000	20	0.000	
27-1	26.02	1.17	0.0384	178.397	0.0384	0.0000	0.000	20	0.000	0.014
27-2			0.0367	178.397	0.0365	0.0002	0.972	20	0.024	
27-3			0.0478	178.397	0.0476	0.0002	0.746	20	0.019	
28-1	5.78	4.68	0.0394	178.397	0.0394	0.0000	0.000	20	0.000	0.011
28-2			0.0447	178.397	0.0449	-0.0002	-0.798	20	-0.020	
28-3			0.0675	178.397	0.0670	0.0005	1.321	20	0.033	
29-1	11.57	2.34	0.0488	178.397	0.0487	0.0001	0.366	20	0.009	0.017
29-2			0.0780	178.397	0.0777	0.0003	0.686	20	0.017	
29-3			0.0718	178.397	0.0714	0.0004	0.994	20	0.025	
30-1	8.67	3.51	0.0411	178.397	0.0411	0.0000	0.000	20	0.000	0.019
30-2			0.0593	178.397	0.0589	0.0004	1.203	20	0.030	
30-3			0.0692	178.397	0.0688	0.0004	1.031	20	0.026	
31-1	0.00	11.71	0.0414	178.397	0.0412	0.0002	0.862	20	0.022	0.007
31-2			0.0535	178.397	0.0548	-0.0013	-4.335	20	-0.108	
31-3			0.0632	178.397	0.0635	-0.0003	-0.847	20	-0.021	
32-1	0.00	0.00	0.0546	178.397	0.0545	0.0001	0.327	20	0.008	0.015
32-2			0.0608	178.397	0.0603	0.0005	1.467	20	0.037	
32-3			0.0565	178.397	0.0565	0.0000	0.000	20	0.000	

number	c(HF) [mol/l]	c($HClO_3$) [mol/l]	m_0 [g]	d_0 [µm]	m_1 [g]	Δm [g]	Δd [µm]	t [min]	r [µm min^{-1}]	$\bar{r}$ [µm min^{-1}]
33-1	0.00	8.19	0.0801	178.397	0.0807	-0.0006	-1.336	20	-0.033	0.016
33-2			0.0550	178.397	0.0544	0.0006	1.946	20	0.049	
33-3			0.0717	178.397	0.0717	0.0000	0.000	20	0.000	
34-1	0.00	3.51	0.0597	178.397	0.0602	-0.0005	-1.494	20	-0.037	0.004
34-2			0.0716	178.397	0.0714	0.0002	0.498	20	0.012	
34-3			0.0635	178.397	0.0642	-0.0007	-1.967	20	-0.049	
35-1	8.67	0.00	0.0591	178.397	0.0595	-0.0004	-1.207	20	-0.030	0.000
35-2			0.0602	178.397	0.0602	0.0000	0.000	20	0.000	
35-3			0.0557	178.397	0.0558	-0.0001	-0.320	20	-0.008	
36-1	20.24	0.00	0.0580	178.397	0.0585	-0.0005	-1.538	20	-0.038	0.000
36-2			0.0627	178.397	0.0633	-0.0006	-1.707	20	-0.043	
36-3			0.0628	178.397	0.0633	-0.0005	-1.420	20	-0.036	
37-1	2.89	1.17	0.0510	178.397	0.0508	0.0002	0.700	20	0.017	0.013
37-2			0.0605	178.397	0.0605	0.0000	0.000	20	0.000	
37-3			0.0638	178.397	0.0635	0.0003	0.839	20	0.021	
38-1	14.46	1.17	0.0400	178.397	0.0399	0.0001	0.446	20	0.011	0.019
38-2			0.0656	178.397	0.0652	0.0004	1.088	20	0.027	
38-3			0.0597	178.397	-	-	-	20	-	
39-1	2.89	7.02	0.0556	178.397	0.0558	-0.0002	-0.642	20	-0.016	0.000
39-2			0.0575	178.397	0.0581	-0.0006	-1.862	20	-0.047	
39-3			0.0410	178.397	0.0415	-0.0005	-2.176	20	-0.054	
40-1	2.89	4.68	0.0483	178.397	0.0482	0.0001	0.369	20	0.009	0.006
40-2			0.0558	178.397	0.0560	-0.0002	-0.639	20	-0.016	
40-3			0.0537	178.397	0.0536	0.0001	0.332	20	0.008	

number	c(HF) [mol/l]	c($HClO_3$) [mol/l]	m_0 [g]	d_0 [µm]	m_1 [g]	Δm [g]	Δd [µm]	t [min]	r [µm min^{-1}]	$\bar{r}$ [µm min^{-1}]
41-1	28.92	0.00	0.0495	178.397	0.0494	0.0001	0.360	20	0.009	0.003
41-2			0.0553	178.397	0.0555	-0.0002	-0.645	20	-0.016	
41-3			0.0510	178.397	0.0513	-0.0003	-1.049	20	-0.026	
42-1	17.35	1.17	0.0623	178.397	0.0622	0.0001	0.286	20	0.007	0.008
42-2			0.0485	178.397	0.0487	-0.0002	-0.736	20	-0.018	
42-3			0.0534	178.397	0.0532	0.0002	0.668	20	0.017	
43	2.89	10.54	0.0679	180.382	0.0678	0.0001	0.266	9770	0.000	0.000
44	8.67	8.19	0.0576	180.382	0.0575	0.0001	0.313	9772	0.000	0.000
45	8.67	3.51	0.0677	178.397	0.0680	-0.0003	-0.791	9700	0.000	0.000
46	11.57	2.34	0.0487	178.397	0.0485	0.0002	0.733	9700	0.000	0.000

For the calculation of the average etching rate $\bar{r}$ of the different etching solutions, the negative etching rates were set to the value 0.000 µm min^{-1}, as no negative etching rates can occur, because no substances are contained in the etching solution that can be deposited on the wafer surface.
